# Advanced strategies for development of vaccines against human bacterial pathogens

**DOI:** 10.1007/s11274-021-03021-6

**Published:** 2021-03-22

**Authors:** Abhinay Sharma, Pooja Sanduja, Aparna Anand, Pooja Mahajan, Carlos A. Guzman, Puja Yadav, Amit Awasthi, Emanuel Hanski, Meenakshi Dua, Atul Kumar Johri

**Affiliations:** 1 School of Life Sciences, Jawaharlal Nehru University, Aruna Asaf Ali Marg, New Delhi, 110067 India; 2 School of Environmental Sciences, Jawaharlal Nehru University, Aruna Asaf Ali Marg, New Delhi, 110067 India; 3 Department of Vaccinology, Helmholtz Centre for Infection Research, Inhoffenstraße 7, 38124 Braunschweig, Germany; 4 Department of Microbiology, Central University of Haryana, Mahendragarh, Harayana India; 5 Department of Microbiology and Molecular Genetics, The Institute for Medical Research, Israel-Canada (IMRIC), Faculty of Medicine, The Hebrew University of Jerusalem, 9112102 Jerusalem, Israel; 6 Translational Health Science and Technology Institute, Faridabad—Gurgaon Expressway, PO box #04, NCR Biotech Science Cluster, 3rd Milestone, Faridabad, Haryana 121001 India

**Keywords:** Genomics, Human pathogens, Protein microarray, Proteomics, Reverse vaccinology, Vaccine

## Abstract

Infectious diseases are one of the main grounds of death and disabilities in human beings globally. Lack of effective treatment and immunization for many deadly infectious diseases and emerging drug resistance in pathogens underlines the need to either develop new vaccines or sufficiently improve the effectiveness of currently available drugs and vaccines. In this review, we discuss the application of advanced tools like bioinformatics, genomics, proteomics and associated techniques for a rational vaccine design.

## Introduction

Emerging literature on the development of drug resistance against pathogens compel us to establish and reinforce the fact that immunization is one of the most effective way to provide long-lasting protection against microbial diseases (Jansen et al. 2018). Despite the availability of various vaccines in the market against different pathogens, there is always a scope in improving already available or preparing new vaccines against pathogens. For example, “first generation vaccines” [use of inactivated or live attenuated pathogens e.g., Bacillus Calmette Guerin (BCG), plague, pertussis, polio, rabies, and smallpox] were developed long ago while “second generation” vaccines (cell components e.g., polysaccharides or protein antigens of the microorganisms and referred as subunit vaccines) were developed in the last decades. Parallel to these vaccine candidates, use of genetic material (DNA and RNA), genetically modified cells and non-virulent viruses packaged with antigenic DNA or RNA are being explored for vaccine development. After learning from first and second generation of vaccine and evolution of advanced tools in vaccine science, there is enormous scope to improve the development of “third generation” with the help of bioinformatics, genomics, proteomics and other associated techniques. Due to several limitations in conventional approaches, it is essential to adapt well proven and advanced techniques to accelerate vaccine development program bypassing time and energy (Rappuoli 2004; Bagnoli et al. 2011).

Current emphasis is focused on the identification of genes/proteins of pathogens which plays important roles in host-pathogen interaction, bacterial pathogenesis and its survival in host body. The last decades have witnessed a number of technological advances that have the potential to be exploited in the expansion of new vaccines development; therefore, the use of these techniques to develop vaccines against important human pathogens is the focus of this article.

## Genomics approaches

Being advantageous over conventional methods, genomics approaches can be used in case of both cultivable as well as non-cultivable microorganisms and are highly efficient to identify all possible antigens expressed universally in different pathogenic strains. Genome-based technologies are helpful to find genuine antigens as well as mimetic antigens that can induce protective immunity against bacterial epitopes (Seib et al. 2012). Gene-expression microarray resolves the problem of complexity of overwhelming data generated by many genomics techniques and offers a useful snapshot of the main cellular events that contribute to the process of microbial pathogenesis, and the identification of key potential vaccine candidate.

Comparative genomics and pan-genome analysis allow a deep comprehensive study of intra and interspecies antigen variability and distribution; and has been described in case of many pathogens like Plasmodium, group B streptococcus (GBS), *Neisseria meningitidis* serogroup B (MenB) and *Streptococcus pyogenes* (Vernikos et al. 2015; Swapna and Parkinson 2017; Carlton et al. 2008; Margarit et al. 2009; McCarthy et al. 2018; Lin et al. 2018; Tettelin et al. 2002; Maione et al. 2005). Intra-species analysis helps by sorting common antigens present in all prevalent strains to act as a universal vaccine candidate. The comprehensive methods like multigenome analysis or pan-genome approach have been demonstrated to identify potential vaccine candidates against highly variable pathogens like *S. pyogenes* (Sharma et al. 2013). The development of a GBS vaccine to fight invasive neonatal disease is considered priority by global health authorities (Lin et al. 2018). The genome sequences of GBS serotype type III strain NEM316 and GBS serotype type V strain 2603 V/R have been exploited to identify novel and universally accepted vaccine candidates (Glaser et al. 2002; Tettelin et al. 2002, 2005; Maione et al. 2005). Huge intra-species diversity suggests that single genome sequence is not entirely representative and does not offer a complete picture of the genetic variability of a species. Therefore, comparative genomics allows identification of potential antigens on the basis of sequence conservation in different serotypes and strains of a given pathogen (Sharma et al. 2013).

Inter-species analysis guides us to identify and filter out antigens that show high degree of similarity with genes present in the human microbial flora avoiding undesirable cross-reaction of vaccine-elicited antibodies against known benign commensal species. An inter-species comparison of predicted protein sets of *S. agalactiae*, *S. pyogenes*, and *S. pneumoniae* has shown that approximately 50 % of proteins are homologous, signifying substantial overlap in potentially relevant pathogenic mechanisms (Tettelin et al. 2002). This information may be exploited to develop a vaccine against multiple species of streptococci. Genomics approaches can identify potential vaccine candidate genes when the pathogen is grown in isolation. Functional aspect of genome dynamics of microorganisms needs to be analysed when they interact with the host. Several functional genomics approaches have been developed to compensate this limitation.

To understand the mechanism of microbial pathogenesis, it is essential to identify the set of genes responsible for the initiation and maintenance of an infection. Initially, there was a lack of suitable techniques for the testing of individual mutants in animal models to understand the function of the genes during infection. The first group of techniques including In vivo expression technology (IVET) and differential fluorescence induction (DFI) has been used to identify bacterial genes by specifically inducing promoters in the infected host (Mahan et al. 1993; Bumann and Valdivia 2007; Adams and Jewett 2018; Roberfroid et al. 2016). In contrast, signature-tagged mutagenesis (STM) was designed to identify genes which are essential for the bacteria to survive in vivo. The technique is based on random mutagenesis of bacteria to identify genes required for in vivo survival where every mutant carries effective molecular signature which can be identified through hybridization (Hensel et al. 1995). The tags from a mixed population of bacterial mutants representing the inoculum and bacteria recovered from infected hosts are detected by PCR, radiolabelling and hybridization analysis. STM is advantageous over other approaches that rely on in vitro grown bacteria and are likely to miss important protective antigens which functions only in vivo (Hensel et al. 1995; Mazurkiewicz et al. 2006; Saenz and Dehio 2005; Ponnusamy et al. 2015). The second group of techniques, including gene expression microarrays, add further advantage to directly measure the gene expression levels on a true genome-wide scale. However, the application of these techniques for analysis of bacterial pathogens during the infection process is still in its early stages. Another method, Reverse Transcriptase Polymerase Chain Reaction (RT-PCR) has qualities that bridge with other methods allowing accurate gene expression measurement on a sub-genomic scale. However, it is difficult to study bacterial pathogenesis during the infection process by these techniques.

An anti-genomic approach—combining genome with serological antigen identification technologies have been used to explore the antigenic repertoire of bacterial pathogens (Meinke et al. 2005; Etz et al. 2002; Fritzer et al. 2010; Nafarieh et al. 2017). This integrated approach is useful for antigen validation, as selected clones can be used directly for the generation of specific immune sera without the demanding task of high throughput recombinant protein production. These sera can be used in surface protein localization studies and in vitro functional assays. The anti-genome of a particular pathogen, defined by this method, typically consists of approximately 100 antigens; most of them are located on the cell surface or secreted into the external environment. Small-insert genomic libraries are also employed for the in vitro protein selection method termed as ‘ribosome display’. It has been employed to identify and characterize the proteins of immunological importance on a genomic scale of the human pathogens (Weichhart et al. 2003; Xiao et al. 2011). In addition, ‘Lambda phage display’ has been used for domain mapping, antigen discovery and protein interactions to identify potential antigens (Nicastro et al. 2014).

Another antibody-based selection method, in vivo induced antigen technology (IVIAT), has been used to identify antigens in pathogens that are only expressed during human infection (Mahan et al. 1993; Hang et al. 2003; Lombardo et al. 2007). For example, *Escherichia coli* expressed genomic libraries of *Vibrio cholerae* were probed by colony blotting, using convalescent human sera. A major challenge in the development of efficient screening methods is the direct selection for protective candidates. For direct selection of protective candidates, expression library immunization technology (ELI) has been successfully applied in case of *Mycobacterium tuberculosis*. This strategy is based on immunization with plasmid DNA incorporating the whole genome split into small fragments. However, ELI is limited to the study of genes that can be expressed in eukaryotic cells, and it also demands animal models which are suitable for screening purposes (Barry et al. 2004; Talaat and Stemke-Hale 2005; Yang et al. 2017).

## Bioinformatics and computational approaches

The genome sequences are complete inventory of every possible vaccine candidate. Advanced bioinformatics tools can be used to examine the genetic content as well as transcription and translation profiles of any pathogen to unravel more details of its pathogenicity. During last two decades after first bacterial (*Haemophilus influenzae*) genome sequence was published, 1000s of bacterial and viral genome sequences have been completed (http://www.ebi.ac.uk/genomes/bacteria.html, http://www.genomesonline.org/cgi-bin/GOLD/bin/sequencing_status_distribution.cgi, http://www.ncbi.nlm.nih.gov/genomes/GenomesGroup.cgi?taxid=10239&opt=Virus). The generated genomic information is used to screen the inclusive set of potential proteins encoded by pathogens for the search of vaccine candidates—an approach referred to as reverse vaccinology (Bagnoli et al. 2011).

In silico whole-genome analysis tools accelerate the process of vaccine candidate identification by integrated use of genomics and proteomics study because one can also predict (I) subcellular localization of vaccine candidate proteins, (II) conserved vaccine candidates among different strains and species, (III) topology of surface proteins, (IV) immunogenicity of different epitopes in vaccine candidate proteins, (V) allergic property of proteins and (VI) 3D structure of vaccine candidate proteins to analyse accessibility of immunologically relevant epitopes with the help of already available bioinformatics tools (Gourlay et al. 2017; Alvarez et al. 2018). Successful use of the bioinformatics tools has been evidently reported in case of many bacterial and viral pathogens. For example, due to the heterogeneous distribution of group A streptococcus (GAS) (> 200 serotypes have been reported) in world population and variation of amino acid sequences in proteins across all serotypes, it is very difficult to identify universal vaccine candidates. Therefore, a comprehensive in silico study has been reported (Sharma et al. 2013). The in silico approaches help to predict of functionality of a particular gene which needs to be verified by using proteomics and genomics tools (Table 1).

**Table 1:** Some important available bioinformatics tools used for data mining and prediction of potential vaccine candidates

Category of tool	Bioinformatics tool	Description	Web address	Reference
General tools	BLAST	Basic Local Alignment Search Tool (BLAST)	http://www.ncbi.nlm.nih.gov/BLAST/	
	PSORTb	For predicting the location of proteins in Gram−negative bacteria (cytoplasm, cytoplasmic membrane, periplasm, and outer membrane or extracellular space)	http://www.psort.org/psortb/	Gardy et al. (2003)
	SignalP	Predicts the presence and location of signal peptidase I (SPaseI) cleavage sites within the N−terminal 70 amino acids of secreted proteins	http://www.cbs.dtu.dk/services/SignalP/	Petersen et al. (2011)
	EXPASY	Analysis of protein sequences and structures	http://www.expasy.org/	
	PDB	Archive contains structures of proteins, nucleic acids and complex assemblies	http://www.pdb.org/pdb/home/home.do	Bernstein et al. (1977)
	PVS	Web server for protein sequence variability analysis tuned to facilitate conserved epitope discovery	http://imed.med.ucm.es/PVS/	Garcia−Boronat et al. (2008)
	AlgPred	Prediction of allergenic proteins	http://www.imtech.res.in/raghava/algpred/	Saha and Raghava (2006)
	CTL PRED	Predicts CTL epitopes that are crucial in vaccine design	http://www.imtech.res.in/raghava/ctlpred/	Bhasin and Raghava (2004)
T-cell epitope prediction	EPIMHC	A curated database of MHC ligands	http://bio.dfci.harvard.edu/epimhc/	Reche and Reinherz (2005)
	PEPVAC	This program fully covers multi−epitope vaccines based on genome wide predictions of MHC-I epitopes	http://immunax.dfci.harvard.edu/PEPVAC/	Reche and Reinherz (2005)
	RANKPEP	Predicts peptide binders to MHC−I and MHC−II molecules	http://bio.dfci.harvard.edu/Tools/rankpep.html	Reche et al. (2004)
	SYFPEITHI	A database containing thousands of peptide sequences known to bind class I and II MHC molecules	http://www.syfpeithi.de/	Rammensee et al. (1999)
	BCIPEP	This database has a collection of the peptides having a role in humoral immunity	http://www.imtech.res.in/raghava/bcipep/index.html	Saha et al. (2005)
	IEDB	This program contains data related to antibody and T−cell epitopes	http://www.immuneepitope.org/	Vita et al. (2010)
	NetCTL 1.2 Server	Predicts CTL epitopes in protein sequences	http://www.cbs.dtu.dk/services/NetCTL/	Larsen et al. (2007)
	MHCPred	MHC I and MHC II binding prediction	http://www.ddg-pharmfac.net/mhcpred/MHCPred/	Guan et al. (2003)
	NetMHC 3.0	Predicts binding of peptides to a number of different HLA alleles	http://www.cbs.dtu.dk/services/NetMHC/	Lundegaard et al. (2008)
	DiscoTope 1.2 Server	Predicts discontinuous B−cell epitopes from three−dimensional protein structures	http://www.cbs.dtu.dk/services/DiscoTope/	Haste Andersen et al. (2006)
	SVMHC	MHC-I and MHC-II binding prediction	https://abi.inf.uni-tuebingen.de/Services/SVMHC	Dönnes and Kohlbacher (2006)
	EpiJen	MHC I binding prediction	http://www.ddg-pharmfac.net/epijen/EpiJen/	Doytchinova et al. (2006)
	MHCBN	A database of MHC binding and non−binding peptides	http://www.imtech.res.in/raghava/mhcbn/	Bhasin et al. (2003)
	PREDEPP	MHC−I epitope prediction software	http://margalit.huji.ac.il/	Schueler−Furman et al. (2000)
	EpiMatrix	EpiVax’s commercial epitope prediction platform	http://www.epivax.com/immunogenicity−screening/epimatrix/	
B-cell epitope tools	BepiPred 2.0	Prediction of linear B−cell epitopes	http://www.cbs.dtu.dk/services/BepiPred/	Jespersen et al. (2017)
	Bcepred	Prediction of linear B−cell epitopes using physicochemical properties	http://crdd.osdd.net/raghava/bcepred/	Saha and Raghava (2004)
	BEST	Prediction of linear B−cell epitopes using	http://biomine.cs.vcu.edu/datasets/BEST/	Gao et al. (2012)
	COBEpro	Prediction of linear B−cell epitopes	http://scratch.proteomics.ics.uci.edu/	Sweredoski and Baldi (2009)
	CBTOPE	Prediction of discontinuous B−cell epitopes	http://crdd.osdd.net/raghava/cbtope/submit.php	Ansari and Raghava (2010)
	DiscoTope 2.0	Prediction of discontinuous B−cell epitopes	http://www.cbs.dtu.dk/services/DiscoTope/	Kringelum et al. (2012)
	EpiPred	Prediction of discontinuous B−cell epitopes	http://opig.stats.ox.ac.uk/webapps/sabdabsabpred/EpiPred.php	Krawczyk et al. (2014)
	Pepitope	Prediction of linear and discontinuous B−cell epitopes using Pepsurf or Mapitope algorithm	http://pepitope.tau.ac.il/	Mayrose et al. (2007)
	Epitopia	Prediction of linear and discontinuous B−cell epitopes	http://epitopia.tau.ac.il/	Rubinstein et al. (2009)

## Proteomics approaches

To overcome the limitations of genomics approaches like (1) mRNA expression levels does not represent the actual amount of active protein in a cell, (2) gene sequence gives incomplete information about post-translational modifications, and (3) genome information does not describe dynamic cellular processes; proteomics has been used in different ways to identify novel vaccine candidates against several human pathogens (Scoffone et al. 2020; Sousa et al. 2020; Sharma et al. 2013; Rodriguez-Ortega et al. 2006; Nilsson et al. 2018; Zielke et al. 2016; Couto et al. 2016; Lo et al. 2017). The present focus on using proteomics is to identify surface proteins in pathogens that can be targeted as potential vaccine candidates. Authors have analysed and identified microbial surface proteins by using two-dimensional gel electrophoresis (2-DE) coupled with mass spectrometry (MS) and matrix-assisted laser desorption/ionization time-of-flight mass spectrometry (MALDI-TOF-MS). However, proteins of hydrophobic nature can not be identified efficiently by this method. Subsequently, a two-dimensional liquid chromatography coupled with tandem mass-spectrometry (2D-LC-MS/MS) was introduced and found to be particularly useful to identify proteins that are either highly hydrophobic or basic, inadequately expressed, with high molecular weight, and extreme isoelectric points (Bagnoli et al. 2011; Gygi et al. 1999; Chen et al. 2006). Improved protocol was adopted to identify surface proteins (surfome) with minimized contamination of cytoplasmic proteins by careful surface digestion of live bacteria with different proteases and mass spectrometry analysis (Rodriguez-Ortega et al. 2006). The same techniques mentioned above have been applied for the analysis of bacterial culture supernatants to identify and analyse bacterial ‘secretome’ (He et al. 2015; Ravipaty and Reilly 2010). Proteomic studies have been used to study the role of the environment in regulating the pathophysiology of several microorganisms as well as investigate host–microbial interactions (Chen et al. 2016; Agudo et al. 2004; Hardwidge et al. 2004). The application of proteomics provides major opportunities to elucidate disease mechanisms and identify new and globally useful vaccine candidates against microbial infections. A comparative proteomic approach allows the selection of vaccine candidates based on differential expression in virulent versus non-virulent strains, invasive versus less invasive conditions or colonizing versus invasive strains (Shaw et al. 2002).

For large-scale quantitative protein expression, ‘Shotgun proteomics’ or multidimensional protein identification technology (MudPIT) were devised to identify the proteins expressed in lower abundance which is not possible by 2-D gel electrophoresis (Wu and Yates 2003). However, this method was not found suitable for comparative analysis unless Isotope-coded affinity tags (ICATs) were developed for the detection of proteins expressed in abundance as well as at low levels (Gygi et al. 1999; Guina et al. 2003). A further advantage of the ICAT method is that it is based on post-isolation stable isotope labelling of proteins and is therefore not limited to incompatibility of cells and tissues with metabolic labelling. Because ICAT label binding is limited to cysteine residue only, quantification of cysteine deficient proteins (10–20% in case of bacteria) may not be done. To overcome these troubles, cysteine independent iTRAQ technique was introduced, which uses a set of four isobaric tags comprised of an amine-specific (peptide N-terminus and lysine residues) reactive group, a neutral linker group (28–31 Da mass), and a reporter region (114–117 Da mass) (Snelling et al. 2007). These labels can therefore be used to simultaneously track up to four samples in a single experiment (Choe et al. 2005). Since the tags have the same complete mass, each peak detected in MS represents a single peptide from the combined four samples. MS/MS of each peptide releases the reporter allowing simultaneous quantitation and identification of the peptide. Another method stable isotope labelling with amino acids in cell culture (SILAC) was designed for the quantification of proteins and has been employed on many pathogens to identify vaccine candidates (Ong et al. 2002; Kani 2017; Jang and Kim 2018). This method is similar to those described above, except that cells subjected to different biological conditions are grown in culture in the presence of an essential amino acid with a stable isotopic nucleus (e.g., deuterium). Therefore, one sample could be incubated with an unlabelled amino acid and the test sample incubated with a deuterated form. Because the amino acid is essential, the organism requires it for survival, and through several replication cycles all that particular amino acid will be present in the cells proteins in either unlabelled (control) or deuterated (test) form, allowing true quantitation (Ong et al. 2002).

Quantitative comparison of protein expression in a variety of normal, developmental and disease states to understand highly regulated and critically timed cellular processes occurring inside pathogenic bacteria can further be characterized by monitoring the fate of post-translationally modified (PTM) proteins that have a role in pathogenesis and ultimately assist in identifying a suitable vaccine candidate (Macek et al. 2019). The combination of proteomics and serological analysis developed a new technology naming serological proteome analysis (SERPA) that has been useful in the identification of potential vaccine candidates (Klade 2002). These technologies provide valuable insights into the molecular basis of microbial pathogenesis to identify potential vaccine candidates that otherwise might not have been identified using more conventional methods.

Antibody-profiling technologies, such as protein microarrays have been used to estimate antibody responses to hundreds of recombinant antigens and allow the screening of high-density protein arrays for enzyme–substrate, DNA–protein, and protein-protein interactions (Bensi et al. 2012; Emili and Cagney 2000). Furthermore, long-lasting humoral responses against different pathogens can be analysed for diagnostics, understanding pathogenic mechanisms and for the development of vaccines against bacteria, protozoa and viruses (Zhou et al. 2015; Kempsell et al. 2015; Felgner et al. 2009; Vigil et al. 2011; Crompton et al. 2010; Duke-Cohan et al. 2009; Fernandez et al. 2011). Despite several advantages, some of the limitations with protein microarray are (1) non-recognition of misfolded or multimeric proteins (2) requirement of additional procedures for identification of PTMs or non-protein antigens (3) requirement of expensive fluorescent microarray scanner and sophisticated statistical methods (4) requirement of expensive robotics for printing arrays. Antibody microarray was found to be the most versatile multiplexed immunoassay technology that was used for the exploratory detection and study of protein abundance, function pathways, and potential vaccine /drug targets. Applications of antibody microarray in basic biology and clinical studies have been recently detailed out providing insights into the current trends and future of protein analysis (Chen et al. 2018).

Apart from application of omics-based tools and reverse vaccinology, development of nucleic acid based vaccines have gained attention in last three decades. It combines the positive aspects of live-attenuated vaccines, viral vectors and subunit vaccines. Nucleic acid based vaccines include viral vectors, plasmid DNA (pDNA) and RNA. These vaccines have their own advantages such as (1) induction of both B and T-cell responses; (2) specificity; (3) high stability; (4) economical; (5) no anti-vector immunity. Published research reports show faster progress in nucleic acid based vaccine development against viruses in comparison to bacterial pathogens. Few reports came up showing potential of these vaccines against bacterial pathogens (Maruggi et al. 2017; Budachetri et al. 2020; Silveira et al. 2017).

## Current status of vaccine research against major pathogens

The catastrophic pandemic outbreak of the century by Covid-19 killing 1000s and infected millions in the world warrants global proactive and integrated research and vaccine development program. Other pathogens which causes, AIDS, malaria, tuberculosis, meningitis, dengue are also the major concerns for vaccine research. Most research groups are focused on intra-species conserved surface proteins of bacterial pathogens (GAS, *Streptococcus pneumoniae, M. tuberculosis*), multi-valent vaccine (Dengue virus and GAS), killed or attenuated whole organisms (Poliovirus, *M. tuberculosis*, Dengue virus and *Helicobacter pylori*), and capsular polysaccharide (GBS, Meningococcus and other Gram-positive bacterial pathogens) for vaccine development. The biggest challenge for vaccine development is against highly variable and fast evolving human pathogen like coronaviruses, HIV, Influenza, Ebola and Nipah viruses.

We have witnessed the efficiency of powerful genomics, proteomics and bioinformatics approaches for identification of vaccine candidates in last two decades. However, despite the fast and efficient target identification, scientists are still facing the slow and laborious validation steps, use of animal models for the testing of the vaccine candidates and clinical trials. On the other hand, safety and affordability are the two important factors that must be taken under consideration for the development of modern vaccines. In our opinion, new vaccines should have the highest effectiveness in developed and developing countries. The successful use of multi-genome analysis, screening and use of reverse vaccinology approach to develop a universal vaccine against highly variable pathogen like GAS and GBS may open new vistas for the potential development of universal vaccines for other human pathogens.

## References

[CR1] Adams PP, Jewett MW (2018). Selection of *Borrelia burgdorferi* promoter sequences active during mammalian infection using in vivo expression technology. Methods Mol Biol.

[CR2] Agudo D, Mendoza MT, Castanares C, Nombela C, Rotger R (2004). A proteomic approach to study *Salmonella typhi* periplasmic proteins altered by a lack of the DsbA thiol: disulfide isomerase. Proteomics.

[CR3] Alvarez B, Barra C, Nielsen M, Andreatta M (2018). Computational tools for the identification and interpretation of sequence motifs in immunopeptidomes. Proteomics.

[CR4] Ansari HR, Raghava GP (2010). Identification of conformational B-cell epitopes in an antigen from its primary sequence. Immunome Res.

[CR5] Bagnoli F, Baudner B, Mishra RP, Bartolini E, Fiaschi L, Mariotti P, Nardi-Dei V, Boucher P, Rappuoli R (2011). Designing the next generation of vaccines for global public health. Omics.

[CR6] Barry MA, Howell DP, Andersson HA, Chen JL, Singh RA (2004). Expression library immunization to discover and improve vaccine antigens. Immunol Rev.

[CR7] Bensi G, Mora M, Tuscano G, Biagini M, Chiarot E, Bombaci M, Capo S, Falugi F, Manetti AG, Donato P, Swennen E, Gallotta M, Garibaldi M, Pinto V, Chiappini N, Musser JM, Janulczyk R, Mariani M, Scarselli M, Telford JL, Grifantini R, Norais N, Margarit I, Grandi G (2012). Multi high-throughput approach for highly selective identification of vaccine candidates: the group a streptococcus case. Mol Cell Proteom.

[CR8] Bernstein FC, Koetzle TF, Williams GJ, Meyer EF, Brice MD, Rodgers JR, Kennard O, Shimanouchi T, Tasumi M (1977). The Protein Data Bank. A computer-based archival file for macromolecular structures. J Mol Biol.

[CR9] Bhasin M, Raghava GP (2004). Prediction of CTL epitopes using QM, SVM and ANN techniques. Vaccine.

[CR10] Bhasin M, Singh H, Raghava GP (2003). MHCBN: a comprehensive database of MHC binding and non-binding peptides. Bioinformatics.

[CR11] Budachetri K, Teymournejad O, Lin M, Yan Q, Mestres-Villanueva M, Brock GN, Rikihisa Y (2020). An entry-triggering protein of *Ehrlichia* is a new vaccine candidate against tick-borne human monocytic ehrlichiosis. mBiol.

[CR12] Bumann D, Valdivia RH (2007). Identification of host-induced pathogen genes by differential fluorescence induction reporter systems. Nat Protoc.

[CR13] Carlton JM, Adams JH, Silva JC, Bidwell SL, Lorenzi H, Caler E, Crabtree J, Angiuoli SV, Merino EF, Amedeo P, Cheng Q, Coulson RM, Crabb BS, Del Portillo HA, Essien K, Feldblyum TV, Fernandez-Becerra C, Gilson PR, Gueye AH, Guo X, Kang’a S, Kooij TW, Korsinczky M, Meyer EV, Nene V, Paulsen I, White O, Ralph SA, Ren Q, Sargeant TJ, Salzberg SL, Stoeckert CJ, Sullivan SA, Yamamoto MM, Hoffman SL, Wortman JR, Gardner MJ, Galinski MR, Barnwell JW, Fraser-Liggett CM (2008). Comparative genomics of the neglected human malaria parasite *Plasmodium vivax*. Nature.

[CR15] Chen EI, Hewel J, Felding-Habermann B, Yates JR (2006). Large scale protein profiling by combination of protein fractionation and multidimensional protein identification technology (MudPIT). Mol Cell Proteom.

[CR16] Chen S, Thompson KM, Francis MS (2016). Environmental regulation of Yersinia pathophysiology. Front Cell Infect Microbiol.

[CR110] Chen Z, Dodig-Crnković T, Schwenk JM, Tao SC (2018) Current applications of antibody microarrays. Clin Proteom 15(1):1–15 10.1186/s12014-018-9184-2 PMC5830343 29507545

[CR18] Choe LH, Aggarwal K, Franck Z, Lee KH (2005). A comparison of the consistency of proteome quantitation using two-dimensional electrophoresis and shotgun isobaric tagging in *Escherichia coli* cells. Electrophoresis.

[CR19] Couto N, Martins J, Lourenco AM, Pomba C, Varela C (2016). A Identification of vaccine candidate antigens of *Staphylococcus pseudintermedius* by whole proteome characterization and serological proteomic analyses. J Proteom.

[CR20] Crompton PD, Kayala MA, Traore B, Kayentao K, Ongoiba A, Weiss GE, Molina DM, Burk CR, Waisberg M, Jasinskas A, Tan X, Doumbo S, Doumtabe D, Kone Y, Narum DL, Liang X, Doumbo OK, Miller LH, Doolan DL, Baldi P, Felgner PL, Pierce SK (2010). A prospective analysis of the Ab response to *Plasmodium falciparum* before and after a malaria season by protein microarray. Proc Natl Acad Sci USA.

[CR21] Doytchinova IA, Guan P, Flower DR (2006). EpiJen: a server for multistep T cell epitope prediction. BMC Bioinform.

[CR22] Duke-Cohan JS, Wollenick K, Witten EA, Seaman MS, Baden LR, Dolin R, Reinherz EL (2009). The heterogeneity of human antibody responses to vaccinia virus revealed through use of focused protein arrays. Vaccine.

[CR23] Dönnes P, Kohlbacher O (2006). SVMHC: a server for prediction of MHC-binding peptides. Nucleic Acids Res.

[CR24] Emili AQ, Cagney G (2000). Large-scale functional analysis using peptide or protein arrays. Nat Biotechnol.

[CR25] Etz H, Minh DB, Henics T, Dryla A, Winkler B, Triska C, Boyd AP, Söllner J, Schmidt W, von Ahsen U, Buschle M, Gill SR, Kolonay J, Khalak H, Fraser CM, von Gabain A, Nagy E, Meinke A (2002). Identification of in vivo expressed vaccine candidate antigens from *Staphylococcus aureus*. Proc Natl Acad Sci USA.

[CR26] Felgner PL, Kayala MA, Vigil A, Burk C, Nakajima-Sasaki R, Pablo J, Molina DM, Hirst S, Chew JS, Wang D, Tan G, Duffield M, Yang R, Neel J, Chantratita N, Bancroft G, Lertmemongkolchai G, Davies DH, Baldi P, Peacock S, Titball RW (2009). A *Burkholderia pseudomallei* protein microarray reveals serodiagnostic and cross-reactive antigens. Proc Natl Acad Sci USA.

[CR27] Fernandez S, Cisney ED, Tikhonov AP, Schweitzer B, Putnak RJ, Simmons M, Ulrich RG (2011). Antibody recognition of the dengue virus proteome and implications for development of vaccines. Clin Vaccine Immunol.

[CR28] Fritzer A, Senn BM, Minh DB, Hanner M, Gelbmann D, Noiges B, Henics T, Schulze K, Guzman CA, Goodacre J, von Gabain A, Nagy E, Meinke AL (2010). Novel conserved group A streptococcal proteins identified by the antigenome technology as vaccine candidates for a non-M protein-based vaccine. Infect Immun.

[CR30] Gao J, Faraggi E, Zhou Y, Ruan J, Kurgan L (2012). BEST: improved prediction of B-cell epitopes from antigen sequences. PLoS ONE.

[CR32] Garcia-Boronat M, Diez-Rivero CM, Reinherz EL, Reche PA (2008). PVS: a web server for protein sequence variability analysis tuned to facilitate conserved epitope discovery. Nucleic Acids Res.

[CR33] Gardy JL, Spencer C, Wang K, Ester M, Tusnady GE, Simon I, Hua S, deFays K, Lambert C, Nakai K, Brinkman FS (2003). PSORT-B: improving protein subcellular localization prediction for Gram-negative bacteria. Nucleic Acids Res.

[CR34] Glaser P, Rusniok C, Buchrieser C, Chevalier F, Frangeul L, Msadek T, Zouine M, Couvé E, Lalioui L, Poyart C, Trieu-Cuot P, Kunst F (2002). Genome sequence of *Streptococcus agalactiae*, a pathogen causing invasive neonatal disease. Mol Microbiol.

[CR35] Gourlay L, Peri C, Bolognesi M, Colombo G (2017). Structure and computation in immunoreagent design: from diagnostics to vaccines. Trends Biotechnol.

[CR36] Guan P, Doytchinova IA, Zygouri C, Flower DR (2003). MHCPred: a server for quantitative prediction of peptide-MHC binding. Nucleic Acids Res.

[CR37] Guina T, Purvine SO, Yi EC, Eng J, Goodlett DR, Aebersold R, Miller SI (2003). Quantitative proteomic analysis indicates increased synthesis of a quinolone by *Pseudomonas aeruginosa* isolates from cystic fibrosis airways. Proc Natl Acad Sci USA.

[CR38] Gygi SP, Rist B, Gerber SA, Turecek F, Gelb MH, Aebersold R (1999). Quantitative analysis of complex protein mixtures using isotope-coded affinity tags. Nat Biotechnol.

[CR39] Hang L, John M, Asaduzzaman M, Bridges EA, Vanderspurt C, Kirn TJ, Taylor RK, Hillman JD, Progulske-Fox A, Handfield M, Ryan ET, Calderwood SB (2003). Use of in vivo-induced antigen technology (IVIAT) to identify genes uniquely expressed during human infection with *Vibrio cholerae*. Proc Natl Acad Sci USA.

[CR40] Hardwidge PR, Rodriguez-Escudero I, Goode D, Donohoe S, Eng J, Goodlett DR, Aebersold R, Finlay BB (2004). Proteomic analysis of the intestinal epithelial cell response to enteropathogenic *Escherichia coli*. J Biol Chem.

[CR41] Haste Andersen P, Nielsen M, Lund O (2006). Prediction of residues in discontinuous B-cell epitopes using protein 3D structures. Protein Sci.

[CR42] He Y, Wang H, Chen L (2015). Comparative secretomics reveals novel virulence-associated factors of *Vibrio parahaemolyticus*. Front Microbiol.

[CR43] Hensel M, Shea JE, Gleeson C, Jones MD, Dalton E, Holden DW (1995). Simultaneous identification of bacterial virulence genes by negative selection. Science.

[CR45] Jang WE, Kim MS (2018). SILAC expands its territory to the pathogenic yeast, *Candida albicans*. Proteomics.

[CR46] Jansen KU, Knirsch C, Anderson AS (2018). The role of vaccines in preventing bacterial antimicrobial resistance. Nat Med.

[CR47] Jespersen MC, Peters B, Nielsen M, Marcatili P (2017). BepiPred-2.0: improving sequence-based B-cell epitope prediction using conformational epitopes. Nucleic Acids Res.

[CR48] Kani K (2017). Quantitative proteomics using SILAC. Methods Mol Biol.

[CR49] Kempsell KE, Kidd SP, Lewandowski K, Elmore MJ1, Charlton S, Yeates A, Cuthbertson H, Hallis B, Altmann DM, Rogers M, Wattiau P, Ingram RJ, Brooks T, Vipond R (2015). Whole genome protein microarrays for serum profiling of immunodominant antigens of *Bacillus anthracis*. Front Microbiol.

[CR50] Klade CS (2002). Proteomics approaches towards antigen discovery and vaccine development. Curr Opin Mol Ther.

[CR51] Krawczyk K, Liu X, Baker T, Shi J, Deane CM (2014). Improving B cell epitope prediction and its application to global antibody-antigen docking. Bioinformatics.

[CR52] Kringelum JV, Lundegaard C, Lund O, Nielsen M (2012). Reliable B cell epitope predictions: impacts of method development and improved benchmarking. PLoS Comput Biol.

[CR53] Larsen MV, Lundegaard C, Lamberth K, Buus S, Lund O, Nielsen M (2007). Large-scale validation of methods for cytotoxic T-lymphocyte epitope prediction. BMC Bioinform.

[CR54] Lin SM, Zhi Y, Ahn KB, Lim S, Seo HS (2018). Status of group B streptococcal vaccine development. Clin Exp Vaccine Res.

[CR55] Lo AW, Moriel DG, Phan MD, Schulz BL, Kidd TJ, Beatson SA, Schembri MA (2017). ‘Omic’ approaches to study uropathogenic *Escherichia coli* virulence. Trends Microbiol.

[CR56] Lombardo MJ, Michalski J, Martinez-Wilson H, Morin C, Hilton T, Osorio CG, Nataro JP, Tacket CO, Camilli A, Kaper JB (2007). An in vivo expression technology screen for *Vibrio cholerae* genes expressed in human volunteers. Proc Natl Acad Sci USA.

[CR58] Lundegaard C, Lamberth K, Harndahl M, Buus S, Lund O, Nielsen M (2008). NetMHC-3.0: accurate web accessible predictions of human, mouse and monkey MHC class I affinities for peptides of length 8–11. Nucleic Acids Res.

[CR59] Macek B, Forchhammer K, Hardouin J, Weber-Ban E, Grangeasse C, Mijakovic I (2019). Protein post-translational modifications in bacteria. Nat Rev Microbiol.

[CR60] Mahan MJ, Slauch JM, Mekalanos JJ (1993). Selection of bacterial virulence genes that are specifically induced in host tissues. Science.

[CR61] Maione D, Margarit I, Rinaudo CD, Masignani V, Mora M, Scarselli M, Tettelin H, Brettoni C, Iacobini ET, Rosini R, D’Agostino N, Miorin L, Buccato S, Mariani M, Galli G, Nogarotto R, Nardi-Dei V, Vegni F, Fraser C, Mancuso G, Teti G, Madoff LC, Paoletti LC, Rappuoli R, Kasper DL, Telford JL, Grandi G (2005). Identification of a universal Group B streptococcus vaccine by multiple genome screen. Science.

[CR63] Margarit I, Rinaudo CD, Galeotti CL, Maione D, Ghezzo C, Buttazzoni E, Rosini R, Runci Y, Mora M, Buccato S, Pagani M, Tresoldi E, Berardi A, Creti R, Baker CJ, Telford JL, Grandi G (2009). Preventing bacterial infections with pilus-based vaccines: the group B streptococcus paradigm. J Infect Dis.

[CR64] Maruggi G, Chiarot E, Giovani C, Buccato S, Bonacci S, Frigimelica E, Margarit I, Geall A, Bensi G, Maione D (2017). Immunogenicity and protective efficacy induced by self-amplifying mRNA vaccines encoding bacterial antigens. Vaccine.

[CR65] Mayrose I, Penn O, Erez E, Rubinstein ND, Shlomi T, Freund NT, Bublil EM, Ruppin E, Sharan R, Gershoni JM (2007). Pepitope: epitope mapping from affinity-selected peptides. Bioinformatics.

[CR66] Mazurkiewicz P, Tang CM, Boone C, Holden DW (2006). Signature-tagged mutagenesis: barcoding mutants for genome-wide screens. Nat Rev Genet.

[CR67] McCarthy PC, Sharyan A, Sheikhi Moghaddam L (2018). Meningococcal vaccines: current status and emerging strategies. Vaccines (Basel).

[CR68] Meinke A, Henics T, Hanner M, Minh DB, Nagy E (2005). Antigenome technology: a novel approach for the selection of bacterial vaccine candidate antigens. Vaccine.

[CR69] Nafarieh T, Bandehpour M, Hashemi A, Taheri S, Yardel V, Jamaati H, Moosavi SM, Mosaffa N (2017). Identification of antigens from nosocomial *Acinetobacter baumannii* clinical isolates in sera from ICU staff and infected patients using the antigenome technique. World J Microbiol Biotechnol.

[CR70] Nicastro J, Sheldon K, Slavcev RA (2014). Bacteriophage lambda display systems: developments and applications. Appl Microbiol Biotechnol.

[CR71] Nilsson Bark SK, Ahmad R, Dantzler K, Lukens AK, De Niz M, Szucs MJ, Jin X, Cotton J, Hoffmann D, Bric-Furlong E, Oomen R, Parrington M, Milner D, Neafsey DE, Carr SA, Wirth DF, Marti M (2018). Quantitative proteomic profiling reveals novel *Plasmodium falciparum* surface antigens and possible vaccine candidates. Mol Cell Proteom.

[CR72] Ong SE, Blagoev B, Kratchmarova I, Kristensen DB, Steen H, Pandey A, Mann M (2002). Stable isotope labeling by amino acids in cell culture, SILAC, as a simple and accurate approach to expression proteomics. Mol Cell Proteom.

[CR73] Petersen TN, Brunak S, von Heijne G, Nielsen H (2011). SignalP 4.0: discriminating signal peptides from transmembrane regions. Nat Methods.

[CR75] Ponnusamy D, Fitts EC, Sha J, Erova TE, Kozlova EV, Kirtley ML, Tiner BL, Andersson JA, Chopra AK (2015). High-throughput, signature-tagged mutagenic approach to identify novel virulence factors of *Yersinia pestis* CO92 in a mouse model of infection. Infect Immun.

[CR77] Rammensee H, Bachmann J, Emmerich NP, Bachor OA, Stevanović S (1999). SYFPEITHI: database for MHC ligands and peptide motifs. Immunogenetics.

[CR78] Rappuoli R (2004). From Pasteur to genomics: progress and challenges in infectious diseases. Nat Med.

[CR79] Ravipaty S, Reilly JP (2010). Comprehensive characterization of methicillin-resistant *Staphylococcus aureus* subsp. aureus COL secretome by two-dimensional liquid chromatography and mass spectrometry. Mol Cell Proteom.

[CR80] Reche PA, Glutting JP, Zhang H, Reinherz EL (2004). Enhancement to the RANKPEP resource for the prediction of peptide binding to MHC molecules using profiles. Immunogenetics.

[CR81] Reche PA, Reinherz EL (2005). PEPVAC: a web server for multi-epitope vaccine development based on the prediction of supertypic MHC ligands. Nucleic Acids Res.

[CR82] Roberfroid S, Vanderleyden J, Steenackers H (2016). Gene expression variability in clonal populations: causes and consequences. Crit Rev Microbiol.

[CR83] Rodriguez-Ortega MJ, Norais N, Bensi G, Liberatori S, Capo S, Mora M, Scarselli M, Doro F, Ferrari G, Garaguso I, Maggi T, Neumann A, Covre A, Telford JL, Grandi G (2006). Characterization and identification of vaccine candidate proteins through analysis of the group A Streptococcus surface proteome. Nat Biotechnol.

[CR84] Rubinstein ND, Mayrose I, Martz E, Pupko T (2009). Epitopia: a web-server for predicting B-cell epitopes. BMC Bioinform.

[CR85] Saenz HL, Dehio C (2005). Signature-tagged mutagenesis: technical advances in a negative selection method for virulence gene identification. Curr Opin Microbiol.

[CR87] Saha S, Raghava GPS, Nicosia G, Cutello V, Bentley PJ, Timmis J (2004). BcePred: prediction of continuous B-cell epitopes in antigenic sequences using physico-chemical properties. Artificial Immune Systems. ICARIS 2004.

[CR111] Saha S, Raghava GPS (2006). AlgPred: prediction of allergenic proteins and mapping of IgE epitopes. Nucleic Acids Res.

[CR86] Saha S, Bhasin M, Raghava GP (2005). Bcipep: a database of B-cell epitopes. BMC Genom.

[CR88] Schueler-Furman O, Altuvia Y, Sette A, Margalit H (2000). Structure-based prediction of binding peptides to MHC class I molecules: application to a broad range of MHC alleles. Protein Sci.

[CR89] Scoffone VC, Barbieri G, Buroni S, Scarselli M, Pizza M, Rappuoli R, Riccardi G (2020). Vaccines to overcome antibiotic resistance: the challenge of *Burkholderia cenocepacia*. Trends Microbiol.

[CR90] Seib KL, Zhao X, Rappuoli R (2012). Developing vaccines in the era of genomics: a decade of reverse vaccinology. Clin Microbiol Infec.

[CR91] Sharma A, Arya DK, Sagar V, Bergmann R, Chhatwal GS, Johri AK (2013). Identification of potential universal vaccine candidates against group a streptococcus by using high throughput in silico and proteomics approach. J Proteome Res.

[CR92] Shaw AC, Gevaert K, Demol H, Hoorelbeke B, Vandekerckhove J, Larsen MR, Roepstorff P, Holm A, Christiansen G, Birkelund S (2002). Comparative proteome analysis of *Chlamydia trachomatis* serovar A, D and L2. Proteomics.

[CR93] Silveira MM, Oliveira TL, Schuch RA, McBride AJA, Dellagostin OA, Hartwig DD (2017). DNA vaccines against leptospirosis: a literature review. Vaccine.

[CR94] Snelling WJ, Lin Q, Moore JE, Millar BC, Tosini F, Pozio E, Dooley JS, Lowery CJ (2007). Proteomics analysis and protein expression during sporozoite excystation of *Cryptosporidium parvum* (Coccidia, Apicomplexa). Mol Cell Proteom.

[CR95] Sousa AC, Neiva HP, Izquierdo M, Alves AR, Duarte-Mendes P, Ramalho AG, Marques MC, Marinho DA (2020). Concurrent training intensities: a practical approach for program design. Strength Cond J.

[CR96] Swapna LS, Parkinson J (2017). Genomics of apicomplexan parasites. Crit Rev Biochem Mol Biol.

[CR97] Sweredoski MJ, Baldi P (2009). COBEpro: a novel system for predicting continuous B-cell epitopes. Protein Eng Des Sel.

[CR98] Talaat AM, Stemke-Hale K (2005). Expression library immunization: a road map for discovery of vaccines against infectious diseases. Infect Immun.

[CR100] Tettelin H, Masignani V, Cieslewicz MJ, Eisen JA, Peterson S, Wessels MR, Paulsen IT, Nelson KE, Margarit I, Read TD, Madoff LC, Wolf AM, Beanan MJ, Brinkac LM, Daugherty SC, DeBoy RT, Durkin AS, Kolonay JF, Madupu R, Lewis MR, Radune D, Fedorova NB, Scanlan D, Khouri H, Mulligan S, Carty HA, Cline RT, Van Aken SE, Gill J, Scarselli M, Mora M, Iacobini ET, Brettoni C, Galli G, Mariani M, Vegni F, Maione D, Rinaudo D, Rappuoli R, Telford JL, Kasper DL, Grandi G, Fraser CM (2002). Complete genome sequence and comparative genomic analysis of an emerging human pathogen, serotype V *Streptococcus agalactiae*. Proc Natl Acad Sci USA.

[CR112] Tettelin H, Masignani V, Cieslewicz MJ, Donati C, Medini D, Ward NL, Angiuoli SV, Crabtree J, Jones AL, Durkin AS, DeBoy RT, Davidsen TM, Mora M, Scarselli M, Margarit y Ros I, Peterson JD, Hauser CR, Sundaram JP, Nelson WC, Madupu R, Brinkac LM, Dodson RJ, Rosovitz MJ, Sullivan SA, Daugherty SC, Haft DH, Selengut J, Gwinn ML, Zhou L, Zafar N, Khouri H, Radune D, Dimitrov G, Watkins K, O’Connor KJB, Smith S, Utterback TR, White O, Rubens CE, Grandi G, Madoff LC, Kasper DL, Telford JL, Wessels MR, Rappuoli R, Fraser CM (2005) Genome analysis of multiple pathogenic isolates of Streptococcus agalactiae: implications for the microbial “pan-genome”. Proc Nat Acad Sci 102(39):13950–13955 10.1073/pnas.0506758102 PMC1216834 16172379

[CR101] Vernikos G, Medini D, Riley DR, Tettelin H (2015). Ten years of pan-genome analyses. Curr Opin Microbiol.

[CR102] Vigil A, Chen C, Jain A, Nakajima-Sasaki R, Jasinskas A, Pablo J, Hendrix LR, Samuel JE, Felgner PL (2011). Profiling the humoral immune response of acute and chronic Q fever by protein microarray. Mol Cell Proteom.

[CR103] Vita R, Zarebski L, Greenbaum JA, Emami H, Hoof I, Salimi N, Damle R, Sette A, Peters B (2010). The immune epitope database 2.0. Nucleic Acids Res.

[CR104] Weichhart T, Horky M, Sollner J, Gangl S, Henics T, Nagy E, Meinke A, von Gabain A, Fraser CM, Gill SR, Hafner M, von Ahsen U (2003). Functional selection of vaccine candidate peptides from *Staphylococcus aureus* whole-genome expression libraries in vitro. Infect Immun.

[CR105] Wu CC, Yates JR (2003). The application of mass spectrometry to membrane proteomics. Nat Biotechnol.

[CR106] Xiao D, Yin C, Zhang Q, Li JH, Gong PT, Li SH, Zhang GC, Gao YJ, Zhang XC (2011). Selection and identification of a new adhesion protein of *Cryptosporidium parvum* from a cDNA library by ribosome display. Exp Parasitol.

[CR107] Yang X, Li M, Liu J, Ji Y, Li X, Xu L, Yan R, Song X (2017). Identification of immune protective genes of *Eimeria maxima* through cDNA expression library screening. Parasite Vectors.

[CR108] Zhou F, Xu X, Wu S, Cui X, Fan L, Pan W (2015). Protein array identification of protein markers for serodiagnosis of *Mycobacterium tuberculosis* infection. Sci Rep.

[CR109] Zielke RA, Wierzbicki IH, Baarda BI, Gafken PR, Soge OO, Holmes KK, Jerse AE, Unemo M, Sikora AE (2016). Proteomics-driven antigen discovery for development of vaccines against gonorrhea. Mol Cell Proteom.

